# 1-Methylcyclopropene Delays Browning and Maintains Aroma in Fresh-Cut Nectarines

**DOI:** 10.3390/foods14020185

**Published:** 2025-01-09

**Authors:** Rui Zhang, Ze Miao, Shuang Xie, Jiao Li, Sheng Tao, Yuqian Jiang, Lingling Pang, Lihua Duan, Xihong Li

**Affiliations:** 1 State Key Laboratory of Food Nutrition and Safety, College of Food Science and Engineering, Tianjin University of Science and Technology, Tianjin 300457, China; zhangruine@163.com (R.Z.); miaoze1223@126.com (Z.M.); xs213727@163.com (S.X.); l1021c@163.com (J.L.); t_sheng0310@163.com (S.T.); jiangyuqian@tust.edu.cn (Y.J.); duanlihuaok@126.com (L.D.); 2 College of Biosystems Engineering and Food Science, Zhejiang University, Hangzhou 310058, China; panglingling2015@163.com

**Keywords:** 1-MCP, nectarines, enzymatic browning, antioxidant capacity, volatile compounds

## Abstract

The color and aroma of nectarines experience adverse effects from cutting, resulting in the fast senescence of fruit tissue. Therefore, 1-methylcyclopropene (1-MCP) was used to treat postharvest nectarines before cutting, and its effect on the surface browning and aroma alteration were investigated. The results indicated that 1-MCP restrained the soluble quinone (SQC) accumulation in fresh-cut nectarines by regulating the peroxidase (POD) and polyphenol oxidase (PPO) activities and the metabolism of phenolic compounds. Compared with the control, 1-MCP pre-cutting treatment maintained the ultrastructural integrity of the cell wall in fresh-cut nectarines, which also showed reduced malondialdehyde (MDA) content and reactive oxygen species (ROS) and enhanced the 1,1-Diphenyl-2-Picrylhydrazyl (DPPH) radical scavenging activities. Electronic nose and GC-MS analysis revealed that the aroma profiles presented significant differences in the control and 1-MCP treatment during the storage at 0 °C for 10 days. The browning value of the 1-MCP pre-cutting treatment was 29.95% lower than the control, which prevented the loss of aroma on day 10. The fresh-cut nectarines could still maintain the characteristic flavor, while the flesh maintains its firmness. The 1-MCP pre-cutting treatment improves the sensory and aroma characteristics of fresh-cut fruits, which is beneficial to the preservation of fresh-cut fruits, improves transportation efficiency, and then improves the overall quality and market attractiveness of the fruit.

## 1. Introduction

Fresh-cut fruits and vegetables are ready-to-eat products formed after fresh fruits and vegetables processing, such as cleaning, trimming, peeling, cutting, and then packaging [1]. The freshness, convenience, and nutritional values of fresh-cut products are factors that are critical to consumer satisfaction. Nectarines are rich in vitamins and other nutrients, with a typically juicy and sweet taste [2]. However, compared with fresh nectarines, fresh-cut nectarines suffer from adverse changes in color and aroma, which affect the economic benefits of the product [3]. Therefore, preserving the quality of fresh-cut nectarines and extending shelf life are the main problems that need to be solved [4,5].

The cutting of fresh nectarines damages the cell tissue, and the direct contact between enzymes and substrates in the tissue leads to various physiological and biochemical reactions, such as cell wall decomposition and phenolic substance oxidation. The phenolics can be oxidized by polyphenol oxidase (PPO) and peroxidase (POD) to quinones with the presence of oxygen, resulting in the fast browning of fresh-cut nectarines [6]. The balance of free radical metabolism within the cells is disrupted when fruits are subjected to abiotic stress, leading to an increase in membrane lipid peroxidation [7]. The fresh cut had a certain impact on aroma and flavor simultaneously. Consequently, the preservation methods for fresh-cut nectarines are still being studied. The combination of high-pressure preservation technology and vacuum packaging could inhibit the PPO activity of fresh-cut peaches and be well preserved at 10 °C for 21 days. This method cannot be fully implemented in terms of technology due to greater resource consumption [8]. Pre-cutting heat treatment effectively controlled the tissue softening and browning of nectarines by changing the gas content in the air conditioning package. However, pre-cutting heat treatment decreased the chromaticity values of the slices and caused total carotenoid loss [9]. It has been observed that NO significantly postponed the ethylene release while preserving the peach fruit’s storage quality. Nevertheless, the microtoxicity of NO has led to a lack of widespread acceptance of its use in the preservation of fruits and vegetables [10]. 1-MCP controls transcriptional and metabolic responses to postharvest quality by delaying ripening and preserving nectarines [11]. However, to the best of our knowledge, not much research has been conducted on the effects of 1-MCP on fresh-cut nectarines.

The interaction of 1-MCP and ethylene receptors inhibited the respiration rate and controlled ethylene production [12]. During climacteric fruit ripening, tissue softening, aroma loss, and pigment accumulation are associated with ethylene production [13]. It has been studied that 1-MCP can delay the ripening of fresh-cut fruits and vegetables, including fresh-cut apples [14], fresh-cut pear [15], fresh-cut kiwifruits, and mangoes [16]. 1-MCP inhibits secondary cell wall-related catabolism and reduces free radical generation to maintain cell structural integrity [17]. It has been studied that 1-MCP may have reduced the browning index (BI) of peach tissue through the inhibition of PPO and POD activities [18]. One of the main factors influencing changes in the concentration of nectarines’ volatile chemicals is ethylene. It was suppressed in the early storage period, but the loss of gas components was recovered in the later period [19,20]. In addition, other studies have shown that 1-MCP treatment significantly enhanced the antioxidant content of postharvest nectarine fruit, preserved the integrity of the cellular membrane, and enhanced the fruit’s ability to withstand chilling [21]. Previous studies have shown that 1-MCP can improve the storage quality of nectarines; the majority of these studies focused on the storage performance of the whole fruit, while the storage characteristics of fresh-cut nectarines have not been thoroughly explored.

The study aims to systematically evaluate the color and fragrance changes of fresh-cut nectarines with 1-MCP pre-cutting treatment. We sought to assess the effects of 1-MCP on browning, antioxidant capacity, cell membrane ultrastructure, and volatile organic compounds. The current study’s objective was to gain a better understanding of the regulatory mechanisms through which 1-MCP improves the browning and aroma to provide a reference for the processing technology of fresh-cut nectarine in the future.

## 2. Materials and Methods

### 2.1. Nectarines, Treatment, and Sample Collection

At the maturity stage, nectarines (Prunus persica cv. “Ruiguang 7”) were picked from a nearby orchard in Beijing, China, and on the same day transported to the laboratory of Tianjin University of Science and Technology (Tianjin, China). Fruits of the same size, no mechanical damage, and no pests were selected and put into the cold room for pre-cooling before the next experiment. Nectarines were randomly and equally divided into two groups, one of which was treated with 1.0 μL·L^−1^ 1-MCP (Xianyang Xiqin Biotechnology Co., Ltd., Xianyang, China) and another group without any treatment [22]. The nectarines and 1-MCP were completely immersed in water (1.2 g) and put into a sealed 1 cubic meter plastic large box (1 m × 1 m × 1 m, oxygen transmission rate is 1500–2700 mL ·m^−2^ ·d^−1^ ·0.1 MPa^−1^) at 0 °C for 18 h, as described by Lee et al. [23]. The content of the active ingredient was 0.18%.

The treated and untreated nectarines were cut into 5 mm thick nectarine slices, about 10 g per piece, and each 300 g nectarine was packaged in a PE plastic bag (oxygen transmission rate is 1500–2700 mL ·m^−2^ ·d^−1^·0.1 MPa^−1^, 150 mm × 200 mm, 0.7 mm thick, Heyuan Evergreen Plastic MFG. Co., Ltd., Heyuan, China). The control and treated groups were stored at 0 °C for 10 days. For quality and physiology analysis, 10 fruits per sample, each with three biological replicates, were used as sampling points set on days 0, 2, 4, 6, 8, and 10. The remaining fruit (with peel) was chopped and immediately frozen in liquid nitrogen for further analysis, and then stored at −80 °C.

### 2.2. Browning Index and Sensorial Qualities

The lightness was measured using a chroma-meter (HP-200, Hanpu Photoelectric Technology Co., Ltd., Shanghai, China) on both sides of the nectarine to obtain L*. The lightness was indicated by the L* index, while reddish-greenish values indicated a* and yellowish-bluish values indicated b*. The color value change was represented as BI once the following formula was applied [24].

BI = 100 × (X − 0.31)/0.172(1)

X = (a* + 1.75 L*)/(5.64 L* + a* − 3.012 b*) (2)

Soluble solid content (SSC) was measured with an Abbe’s refractometer (WY060T, North and South Instrument Co., Ltd., Zhengzhou, China). Fruit samples were placed in a mortar, and the juice was extracted by centrifugation (4 °C, 4000× *g*, 10 min).

Ascorbic acid (ASA) was evaluated using the method described by Jiang et al. [11]. Fresh-cut nectarine samples (10 g) were crushed into a pulp, and mixed with a tiny amount of oxalic acid solution (20 g·L^−1^), and the volume was adjusted to 100 mL using oxalic acid. After 10 min of extraction, the mixture was centrifuged for 5 min at 4 °C at 7000× *g*. A 2,6-dichlorophenol indophenol solution was then used to titrate 10 mL of the supernatant. Three iterations of the experiment were conducted.

Sensory quality was evaluated by a panel of 20 professionally trained judges on four dimensions: aroma, taste, texture, and color, with a full weight factor of 0.3, 0.2, 0.2, 0.3 [25]. The four dimensions were categorized into 11 attributes with the following 9 ratings (Table 1): absence of sensation, just recognizable, very weak, weak, slight, moderate, intense, very intense, and extremely intense. Judges were randomly given sample evaluations in bright daylight, and mouthwash was supplied in between assessments.

### 2.3. Enzymatic Activities of PPO, POD, Total Phenolic Content (TPC), and Soluble Quinone Content (SQC)

The activities of PPO and POD were evaluated using the method described by Du et al. [26]. Samples of frozen pulp tissue (0.5 g) stored at −80 °C after treatment with liquid nitrogen were added to acetate sodium acetate buffer (5 mL, pH 5.5, 4% polyvinyl-poly-pyrrolidone, 1% Triton X-100), homogenized, and centrifuged (4 °C, 10,000× *g*, 30 min), which determined the PPO and POD activities during low temperature.

The PPO extract was added to the mixture of phosphate buffer (200 μL, 50 mM, pH 6.5) and catechol solution (50 μL, 50 mM), and the absorbance at 420 nm was recorded as the initial value after 15 s of timing. For six consecutive determinations, the data were recorded at one-minute intervals. The results were expressed as U·kg^−1^ fresh weight, and each experiment was conducted three times. The POD reaction mixture contained 20 μL 500 mM H_2_O_2_, 30 μL of crude enzyme extracts, and 180 μL 25 mM guaiacol. After combining the enzyme extract and guaiacol solution, H_2_O_2_ was added to speed up the reaction. There were fifteen seconds at the start. Six points of data were collected by recording the absorbance value of the reflecting system at a wavelength of 470 nm per minute. Three iterations of the experiment were conducted. The POD activity was expressed as U·kg^−1^ total soluble protein or units of soluble protein per kilogram. One absorbance per minute increase in the sample was counted as one unit of peroxidase activity.

The method of Folin–Ciocalteu with slight modifications was followed [27]. After grinding the 5 g sample in an ice bath, 25 mL of methanol was added, shaken in the dark, sonicated at 4 °C for 30 min, and then centrifuged at 10,000× *g* for 10  min at 4 °C. The SQC was determined by measuring the absorbance of the supernatant at 432 nm [28]. The absorbance of the supernatant mentioned above was read at 437 to determine the SQC, and the result was expressed as A_437_. To assay TPC, the Folin reagent (1 mL, 1  mol ·L^−1^, Shanghai Yuanye Bio-Technology Co., Ltd., Shanghai, China) was homogenized with 0.2 mL supernatant, shaken and reacted in the dark for 10 min, and sodium carbonate solution (1  mL, 1  mol ·L^−1^) was then added. Methanol was used as a blank to read the absorbance value at 765 nm. Gallic acid was used as the standard curve. Gallic acid equivalent (g ·kg^−1^) was used to express the results.

### 2.4. Oxidant Activity and Antioxidant Capacity, Hydrogen Peroxide (H_2_O_2_) Content, Superoxide Anion Radicals (O_2_^•−^) Content, Malondialdehyde (MDA) Content, and 1,1-Diphenyl-2-Picrylhydrazyl (DPPH) Scavenging Ability

According to the method by Diu et al. [26] for MDA determination, trichloroacetic acid solution (TCA, 5 mL, 100 g·L^−1^) was added to 1.0 g of fruit samples, ground well in an ice bath, and then 10,000× *g* centrifugation was performed at 4 °C for 10 min. The mixture of thiobarbituric acid (TBA, 2 mL, 6.7 g·L^−1^) added to the supernatant (2 mL) was boiled for 20 min, and the absorbance value was measured at a 450 nm, 532 nm, and 600 nm wavelength. The micromole per kilogram of fresh weight, μmol·kg^−1^, was used to express the MDA content.

The O_2_^•−^ content was measured using a kit (Shanghai Yuanye Bio-Technology Co., Ltd., Shanghai, China). The O_2_^•−^ concentration was expressed as micromoles per kilogram of fresh weight.

The Sudewi et al. method [29] was followed in order to collect the supernatant for H_2_O_2_ analysis promptly. The content was determined using a kit (Beijing Solarbio Science and Technology Co., Ltd., Beijing, China). The results were expressed as millimoles per kilogram of fresh weight.

The DPPH was measured to determine the radical scavenging activity of the extract utilizing the stable radical DPPH detection followed by Tang et al. [30], with some modifications. The powder of 1, 1-diphenyl-2-picrylhydrazyl was dissolved into absolute ethanol to prepare the DPPH solution (6.5 × 10^−4^ mol·L^−1^), which was stored at a low temperature and protected from light. Different gradients of trolox solution or centrifugation supernatant were added to the DPPH solution and the reaction was carried out at 25 °C in the dark for 4 h. The absorbance was measured at 517 nm. The DPPH radical scavenging activity was expressed as micromoles of trolox equivalents (TEs) per gram of sample (mmol TE·kg^−1^) (r^2^ = 0.999).

### 2.5. Ultrastructure Analysis by TEM (Transmission Electron Microscope)

The ultrastructure of nectarine samples was observed by transmission electron microscopy according to the determination method of Zhou et al. [31], with slight modifications. Freshly cut small nectarines (3 mm × 2 mm × 3 mm) were fixed in glutaraldehyde solution (2.5%) for 2 h before image capture at the testing company (Servicebio Technology Co., Ltd., Wuhan, China). Samples were fixed with 1% (*w*/*v*) osmium tetroxide 0.1 M PBS buffer solution for 2 h and then dehydrated through a graded ethanol series. The dehydrated peach samples were embedded in Epoxy Resin 812 baked at 70 °C for 24 h and then cooled to room temperature. The samples were used to cut ultrathin tissue sections (EM UC7, Leila, Barcelona, Germany). Ultrathin sections (70 nm) were observed in a Hitachi H7650 TEM system (Tokyo, Japan) transmission electronic microscope at 80 kV.

### 2.6. Determination of Volatile Gas Components

A PEN 3 E-nose device (Winmuster Airsense Analytics Inc., Schwerin, Germany) was used to assess volatiles produced by fresh-cut nectarines [32,33]. Two grams of granular fresh sample was transferred to a 20 mL plastic tube. Saturated sodium chloride solution 2 mL was added to homogenize. The samples airproofed with cover gaskets were heated to 40 °C for 20 min. The headspace gas was injected continuously with a rate of 100 mL·min^−1^ during the measurement process. The clean phase and the measurement phase lasted for 80 s and 140 s, respectively. Data at the 110th second of the measurement process were selected for subsequent analysis.

The determination of volatile compounds was accomplished by a solid phase micro extraction (SPME) method using Gas Chromatography–Mass Spectrometry (Thermo Fisher Scientific ISQ 7000, Waltham, MA, USA). A trisplus automated solid phase microextraction (SPME) sampler equipped with a 65 µm PDMS/DVB on PDMS fiber (57310-U, Supelco Co., Bellefonte, PA, USA) was employed for the extraction. The sample (5 g) was ground in an ice bath and transferred to a 20 mL glass vial, and NaCl, ethylene diamine tetraacetic acid (EDTA), and the internal standard 2-octanol (20 μL, 7.90 mg·L^−1^) were added. A 30 m × 0.25 mm × 0.25 µm HP-5 MS (Agilent Technologies, Santa Clara, CA, USA) was fitted to the GC-MS system. As a carrier gas, helium was used at a flow rate of 1.0 mL·min^−1^. Starting at 40 °C, the oven temperature increased by 3 °C per minute to reach 100 °C, and was followed by 5 °C·min^−1^ to reach 245 °C. The ion source was set to 280 °C, the transfer line was moved to 250 °C, and the quadrupole mass detector was modified to 150 °C. The *m*/*z* 35–500 amu was the scan range. The ratio of each compound’s peak regions to the internal standard was used to calculate the relative quantity of the discovered compounds [34].

### 2.7. Statistical Analysis

The completely random principle guided the experiment’s design. All graphs in this study were supplied as the mean ± standard deviation (SD) and produced by Origin 2019 software (OriginLab, Corp., Northampton, MA, USA). The statistical program SPSS26.0 was used to process and analyze the data (IBM, Armonk, NY, USA). To find significant differences (*p*  <  0.05), the least significant difference (LSD) was computed.

## 3. Results

### 3.1. Textural Attributes and Sensory Evaluation of the Overall Quality of Fresh-Cut Nectarines

**Figure 1:**
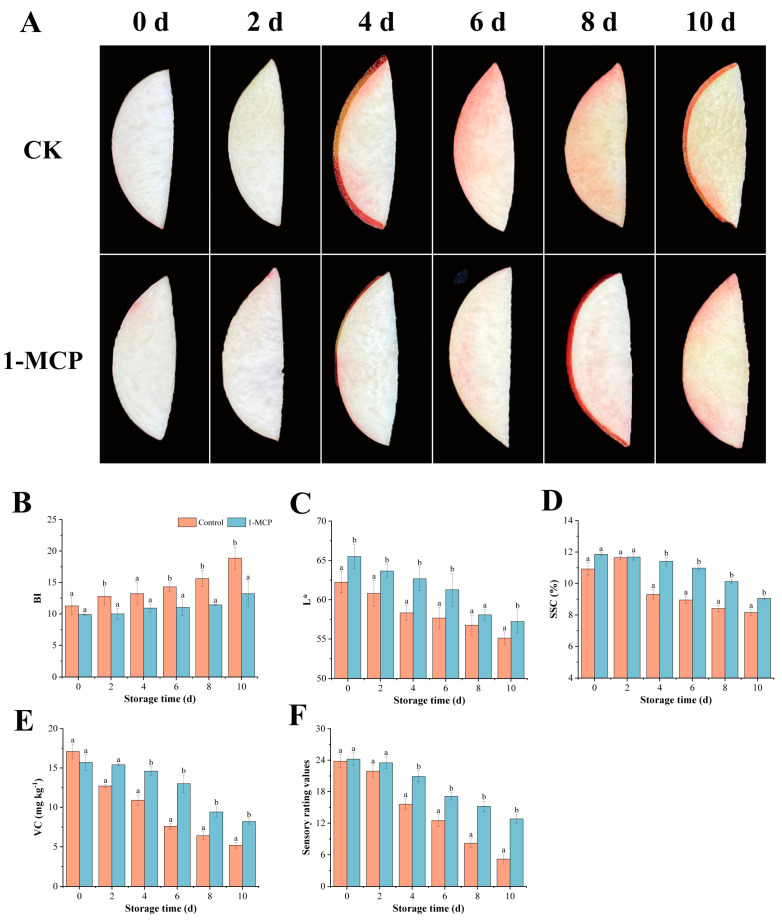
The browning of fresh-cut nectarines treated with 1-MCP during storage. (**A**) The browning; (**B**) BI; (**C**) lightness L*; (**D**) SSC; (**E**) ASA; (**F**) sensory rating values. Standard deviation (*n* = 4) is shown by vertical bars. Different letters denote significant differences between various treatments for each sampling time at *p*  < 0.05.

Browning in fresh-cut fruit and vegetables is primarily indicated by color change, which also affects shelf life and other quality attributes. As shown in Figure 1A, the color of fresh-cut nectarines in 1-MCP and control groups gradually deepened with time. Since day 6, there has been a noticeable color change in the control, but the fresh-cut nectarines treated with 1-MCP still maintained a relatively bright color, even on day 8. As for the BI depicted in Figure 1B, the BI of the control and 1-MCP pre-cutting treatment at day 10 were 1.68 times and 1.33 times higher than day 1, respectively. The BI of the 1-MCP pre-cutting treatment on day 10 was identical to that of the control on day 4, which is consistent with the sensory evaluation results mentioned above. Generally, the browning of the cut surface is accompanied by a decrease in L*. As seen in Figure 1C, the L* values of the 1-MCP pre-cutting treatment and the controls steadily decreased, while the nectarines in the 1-MCP had a greater L* value. This result indicates that the control had more severe browning on the nectarines, while the loss of brightness in the nectarines of the 1-MCP was more minor. Therefore, 1-MCP can effectively control the degree of browning in fresh-cut nectarines, prolong their storage time, and improve their commercial quality.

With the extension of the storage period, the SSC of fresh-out nectarines continued to decrease; the 1-MCP pre-cutting treatment on day 10 was 3.48% lower than day 0, and 2.80% lower in the control group (Figure 1D). ASA content is an important nutrient in the tissue, which represents the quality of freshly cut peaches. There is no significant difference in the content of the first two days, but the content of the control group decreases rapidly with the storage time, and there is a significant difference in the content of the later stage. The ASA of the 1-MCP pre-cutting treatment decreased from 15.7 mg kg^−1^ to 8.2 mg kg^−1^, with a reduction of 47.77%, while the control decreased by 67.70% (Figure 1E).

The sensory analysis results showed that the taste, odor, color, and overall acceptance of fresh-cut nectarines gradually decreased with the extension of the storage time, and the untreated group decreased faster, with the lowest score of 3.2. The total score of peaches in the 1-MCP group decreased from 23.8 to 12.8 (Figure 1F). On day 10, the untreated peach slices had the lowest score, with an imbalance of acidity and sweetness, less juice, and severe browning. On day 8, the 1-MCP group retained the nectarine fragrance, while the flavor remained consistent.

### 3.2. Browning-Related Index

**Figure 2:**
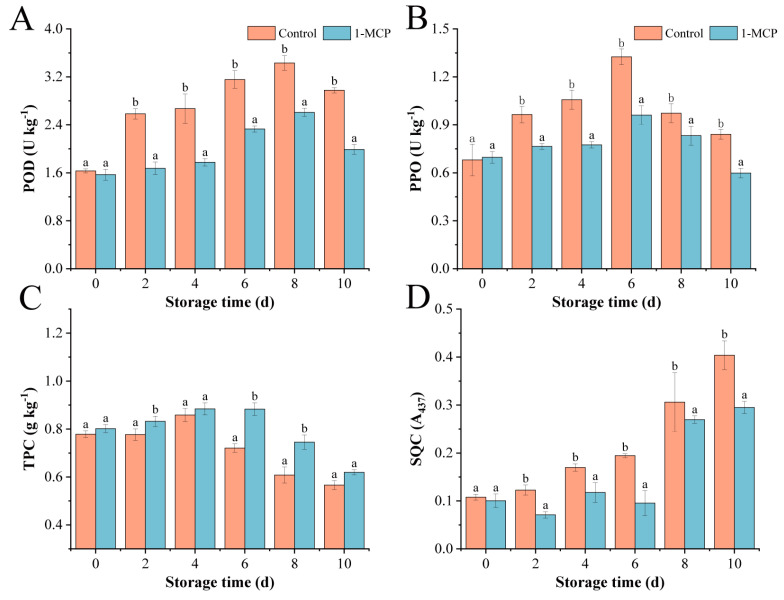
Phenolic metabolism in fresh-cut nectarines treated with 1-MCP. (**A**) PPO; (**B**) POD; (**C**) TPC; (**D**) SQC. Standard deviation (*n* = 3) is shown by vertical bars. Different letters denote significant differences between various treatments for each sampling time at *p*  < 0.05.

To further study the enzymatic browning metabolism, POD activity responds to external stimuli. On day 0, there was a significant difference between the 1-MCP and the control, with the control exhibiting 1.89 times higher POD activity compared to the 1-MCP (Figure 2A), and groups showed a peak value on day 8. The POD content of the 1-MCP decreased considerably in comparison to the control, suggesting that 1-MCP therapy prevented POD activity.

PPO is an important enzyme associated with browning, as it catalyzes the oxidation of phenolic compounds, leading to the formation of dark pigments. Most anti-browning strategies aim to regulate PPO activity by controlling this enzymatic process [35]. PPO activity in the control and 1-MCP exhibited a trend of first rising and then falling (Figure 2B). With the appearance of the peak value on the sixth day, the PPO activity content reached 2 times and 1.2 times that of the first day of fresh-cut nectarines for the control and 1-MCP pre-cutting treatment, respectively, and then decreased. Quinone’s feedback suppression of PPO activity at high doses may be the origin of this behavior. Compared to the control, the 1-MCP had lower PPO activity levels, and the 1-MCP slowed down the rate of increase in PPO activity during the entire storage period.

The browning that occurs during postharvest processing is closely related to PPO in tissues. 1-MCP increased the initial stage of phenolic compound accumulation and stimulation. The TPC revealed a rising trend followed by a declining trend in the control and 1-MCP (Figure 2C), reaching a peak on day 4, respectively, at 0.855 and 0.884. The TPC content of the 1-MCP was slightly higher than that of the control, and decreased to a minimum on day 10, reaching 77% and 73% of the original values, respectively.

Quinone compounds further polymerize to form brown or brownish polymeric compounds [36]. The control and 1-MCP showed an overall upward trend, with contents of 0.11 and 0.10 on the first day, respectively, and rapidly increased to 0.38 from day 6 to day 10 (Figure 2D). Throughout the storage period, the total content of the 1-MCP was marginally less than the control during the storage period. These results confirmed that 1-MCP inhibited the formation of soluble quinones, thereby slowing the browning process in fresh-cut nectarines.

### 3.3. Oxidant Activity and Antioxidant Capacity

The contents of MDA, H_2_O_2_, O_2_^•−^, and DPPH were assayed, which revealed the effects of 1-MCP on peroxidation metabolism. When plants are damaged, they produce a large amount of reactive oxygen species (ROS), including O_2_^•−^ and H_2_O_2_. The accumulation of ROS components can accelerate browning and even destroy the cell membrane structure.

**Figure 3:**
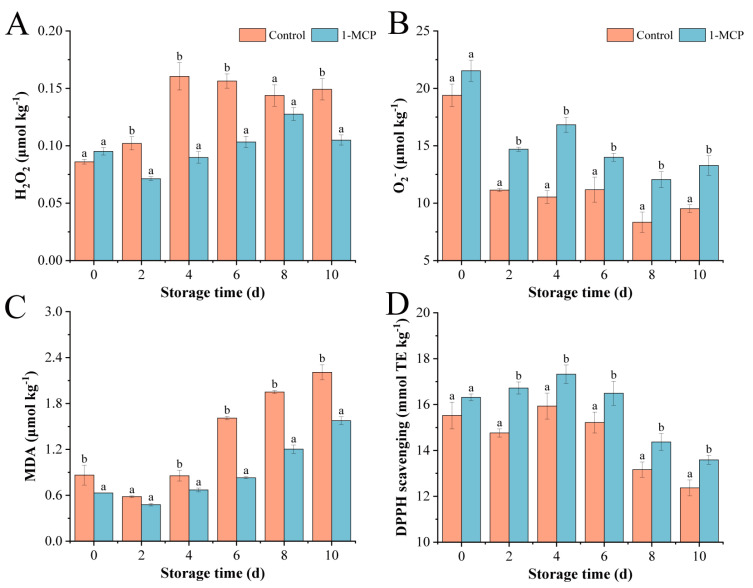
Oxidant activity and antioxidant capacity in fresh-cut nectarines treated with 1-MCP. (**A**) H_2_O_2_; (**B**) O_2_^•−^; (**C**) MDA; (**D**) DPPH. Standard deviation (*n* = 3) is shown by vertical bars. Different letters denote significant differences between various treatments for each sampling time at *p*  < 0.05.

The contents of H_2_O_2_ in the control and 1-MCP revealed a downward trend after an initial upward trend (Figure 3A), reaching peak values on day 4 and day 8, respectively. The 1-MCP pre-cutting treatment delayed the time when the peak H_2_O_2_ content appeared. Moreover, the 1-MCP group’s H_2_O_2_ production was significantly higher than the control after day 1, which demonstrates that the 1-MCP pre-cutting treatment greatly enhanced the production of H_2_O_2_.

In this study, the content of O_2_^•−^ gradually decreased with time (Figure 3B). The content in the control and 1-MCP decreased to 62% and 49% of the initial values on day 10, respectively. The O_2_^•−^ content was lower in the 1-MCP than in the control. This indicates that the 1-MCP pre-cutting treatment effectively inhibits the production of O_2_^•−^ in fresh-cut nectarines.

Under the catalysis of enzymes and reactive oxygen species, the cell membrane can produce MDA [37], which can cause particular damage to the cell membrane and organelles of fruits and vegetables (Figure 3C). MDA reflects the degree of membrane lipid peroxidation. The 1-MCP resulted in lower MDA levels compared to the control, suggesting that 1-MCP effectively decelerated MDA accumulation in fresh-cut nectarines.

The DPPH free radical scavenging rate is a significant indicator of antioxidant capacity and can be used to represent the overall antioxidant potential of tissue [38]. It is worth noting that there is a sure consistency between the trend of DPPH scavenging activity percentage and the content of TPC. The 1-MCP demonstrated higher levels of free radical scavenging capacity in comparison to the control (Figure 3D). This significantly improved the antioxidant capacity and effectively delayed browning in fresh-cut nectarines.

### 3.4. Observation on the Ultrastructure of the Organelles and Cell Membranes

**Figure 4:**
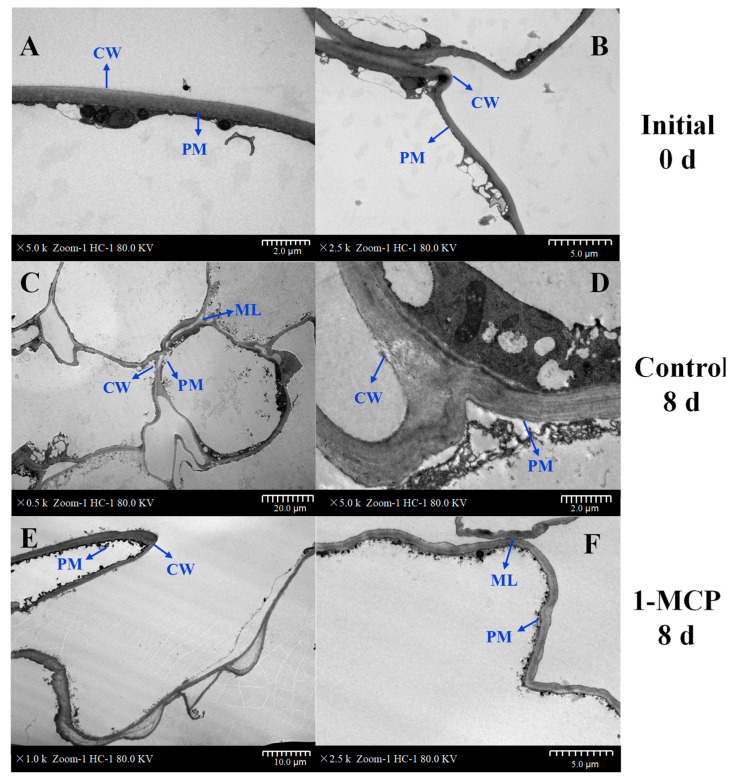
Cell membrane ultrastructure analysis of pulp tissue from fresh-cut nectarines using TEM. Ultrastructure of fresh-cut nectarines on day 0 in untreated fruit (**A**,**B**); ultrastructure of untreated fresh-cut nectarines cells after day 8 at 0 °C (**C**,**D**); treated with 1-MCP at 0 °C on day 8 (**E**,**F**). PM: plasma membrane; CW: cell wall; ML: middle lamella.

In this study, the cell membrane region of the samples was examined using a transmission electron microscope (TEM) on days 0 and 8 (Figure 4). Freshly cut nectarines on day 0 had an intact cell wall structure with distinct cell outlines, a tightly packed central lamella, and abundant intracellular starch granules (Figure 4A,B). The cell layer is lysed, resulting in a split between the intercellular layer and the cells after 8 days of storage (Figure 4C,D). The lack of structural support from the cell wall, along with the weakening cytoplasmic membrane, caused the cells to become deformed, swollen, and irregularly shaped. On the other hand, the morphology of the 1-MCP-treated cells was largely unaltered, and their cell walls showed a high level of continuity and completeness. The middle layer, although thinned, exhibited a well-organized structure (Figure 4E,F). The significant differences in the microstructures of treated and untreated fresh nectarines suggested that the 1-MCP pre-cutting treatment effectively preserved cell membrane integrity.

### 3.5. Regulation on the Volatile Aroma by 1-MCP

**Figure 5:**
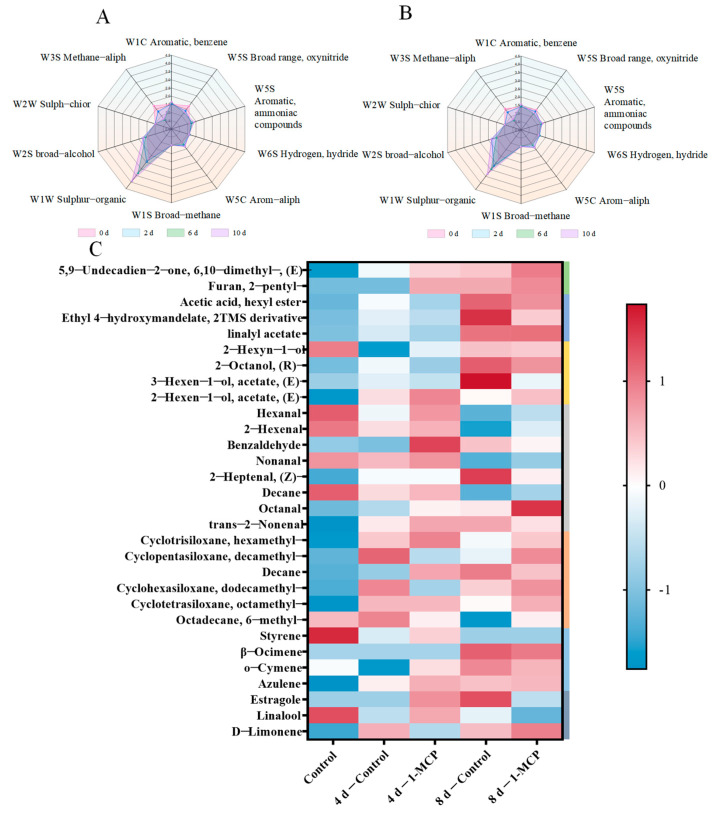
Comparison of volatile compounds in fresh-cut nectarines treated with 1-MCP and the control stored at 0 °C. (**A**) E-nose sensing profiles with the control, (**B**) E-nose sensing profiles with 1-MCP pre-cutting treatment. (**C**) Heatmap volatile organic compounds, and different colored bars represent different types of compounds. Z-score shows the relative contents after normalization processing. The differential content of each compound is represented by the Z-score color scale following normalization.

Minor variations in volatile flavor compounds caused differences in the E-nose sensor response values. E-nose sensing on fresh-cut nectarines’ volatile organic compounds (VOCs) on days 0, 2, 6, and 10 is shown in Figure 5A,B. The responses to S4, S6, and S9 with a generally lower amplitude showed no significant differences, which are, respectively, for hydrogen, broad-methane, and sulph-chlor. The sensor S7 presented higher response values and experienced more significant changes than the other sensors. Compared with the control, the sensor S1 response was decreased in the 1-MCP pre-cutting treatment, which is for broad aromatic compounds. The sensors S8 were more sensitive to the volatile compounds produced by fresh-cut nectarines, indicating the synthesis of compounds with partial aromaticity. Significantly, the sensor W2S (aromatic compounds) displayed a declining trend during the period of 2–10 days.

The volatile composition of fresh-cut nectarines by HS-SPME GC-MS was recorded and analyzed on days 0, 4, and 8 during storage at 0 °C (Figure 5C). Based on the relative content proportions of volatile compounds, 30 volatile compounds were identified in fresh-cut nectarines (Table 2). To better illustrate the differences and patterns among the samples, normalization was performed before generating the heatmap. These compounds included 8 aldehydes, 4 alcohols, 6 alkanes, 4 alkenes, 3 esters, and 3 terpenoids heterocyclic compounds. The types and concentrations of VOCs varied greatly with storage time, with a decrease in aldehydes and an increase in esters, and the overall types and concentrations of VOCs were lower than in control fruits. Overall, aldehydes and alcohols are the predominant compounds, comprising 46% of the species and accounting for 88.42% of the total concentrations, whereas alkanes, alkenes, esters, and other volatile organic compounds make up less than 12% of the concentrations. The content of hexanal accounted for 46.19% on day 0, which was the highest relative content of all aldehydes. The hexanal in the control was reduced by 26.24% on day 8 compared with day 0, while the treatment had only an 18.89% increment. In addition, as the second most important aldehyde compound, 2-Hexenal on day 0 accounted for 37.55% of the total volatiles. The majority of detected free esters increased sharply with fruit senescence, starting at 0.45% on day 0 and increasing twofold on day 8 in the control. In the 1-MCP, the content of free esters was lower in the first four days compared to the control. 1-MCP suppresses the release of volatile esters in the fresh-cut nectarines. More importantly, both changes in terpenoids, such as the content of linalyl acetate and the levels of D-limonene, exhibited a similar trend.

### 3.6. Correlation Analysis

**Figure 6:**
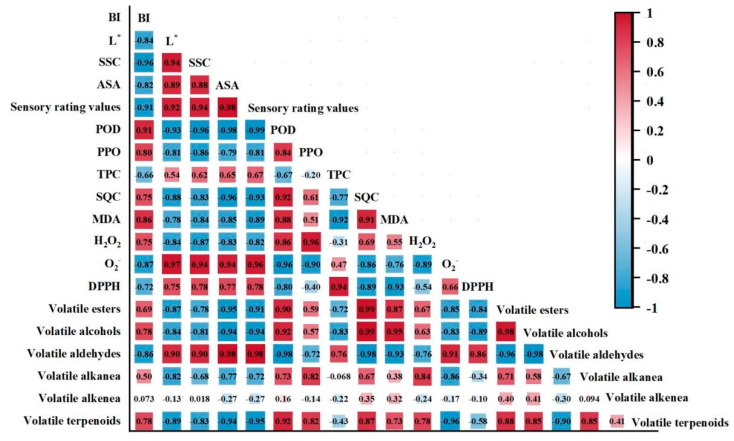
Pearson’s correlation coefficients for surface, antioxidant, membrane peroxidation, and volatile qualities associated with fresh-cut nectarines. The dots’ numbers represent the coefficients; positive and negative correlations are represented by the red and blue colored dots, respectively.

To unveil the potential relationship among browning, antioxidant activities and volatile organic compound content of fresh-cut nectarines during the entire storage at 0 °C, the analysis method employed was Kendall’s correlation analysis (Figure 6). The BI values for surface browning showed a positive correlation with POD, PPO, SQC, MDA, and H_2_O_2_ (0.68 < r^2^ < 0.88), and was negatively correlated with TPC, O_2_^•−^, and DPPH (−0.87 < r^2^ < −0.66). MDA is one of the major membrane lipid peroxidation products, and was negatively correlated with TPC, O_2_^•−^, DPPH, and volatile aldehydes (−0.93 < r^2^ < −0.76), but positively correlated with POD and SQC, as well as volatile esters, alcohols, and terpenoids (0.73 < r^2^ < 0.95). The volatile alkenea degree of correlation with other indicators is lower, which may be due to the accumulation and decomposition of related substances.

## 4. Discussion

The degree of browning on fresh-cut fruit indicates its commercial value and freshness. In this study, 1-MCP inhibited browning on the surface of fresh-cut nectarines and delayed the increase in BI and L* values. The appearances in the photos (Figure 1A) and the color measurements discussed above are all in agreement. The results showed that 1-MCP could effectively slow down the loss in SSC content in fresh-cut nectarines during storage. With the decrease in SSC, cell walls relax and the rate of cellular senescence increases, which was similar to the results of Cai et al. [20]. This effect may be attributed to the ability of 1-MCP to inhibit ethylene production, thereby delaying the postharvest ripening and senescence process of fresh-cut nectarines. Higher ASA during storage also plays an important role in delaying the browning of fruits and vegetables. 1-MCP can reduce ASA loss in fresh-cut nectarines and slow down the oxidative damage after cutting, thereby delaying the deterioration process and thus inhibiting the occurrence of browning [17]. In this study, we found that the 1-MCP pre-cutting treatment moderately slowed down browning during storage while effectively maintaining the sensory quality of fresh-cut nectarines.

**Figure 7:**
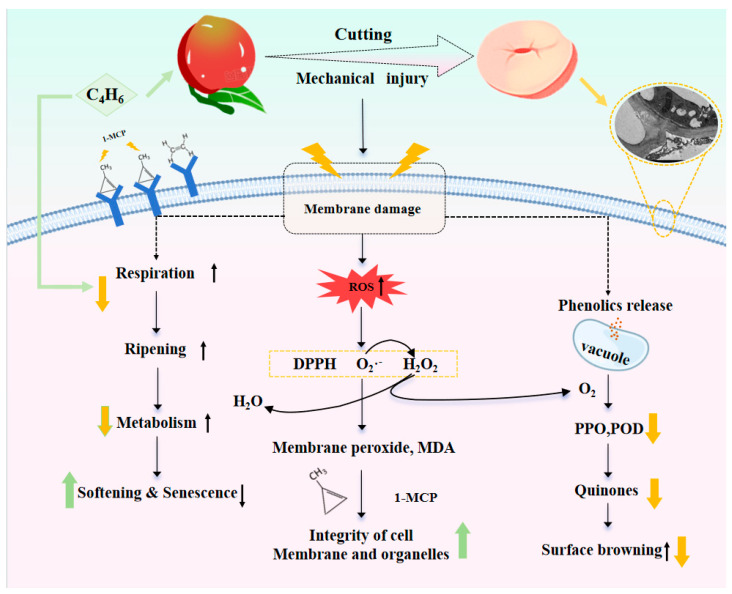
A schematic representation of the 1-MCP’s regulation mechanism for fresh-cut nectarines. Green arrow indicates up and yellow arrow indicates down.

The content of phenolic substances and the activity of phenol oxidase in fruit and vegetable tissues are internal factors affecting the degree of browning, and soluble quinones are the browning products [39]. In this study, the POD activity of 1-MCP was decreased, which slowed ethylene generation and reduced browning. The reduction in browning observed with the 1-MCP pre-cutting treatment may be attributed to the regulated activity of PPO and POD [40]. Similarly, elevated PPO activity enhances the susceptibility of fresh-cut nectarines to browning [41]. Ascorbic acid has been shown to reduce the activity of PPO, and 1-MCP effectively inhibits the reduction in ASA content, thereby mitigating the activity of PPO [42]. When fruits and vegetables are cut, enzyme-catalyzed browning occurs, the normal respiratory chain is disrupted, and contact with air and cutting damage can stimulate polyphenol synthesis in fresh-cut nectarines [43]. 1-MCP enhances the stimulation of this antioxidant system’s stress response, inducing fresh-cut nectarines to increase their antioxidant and stress resistance capabilities, and inhibiting enzyme-catalyzed browning after cutting, which the relationships of different parameters in fresh-cut peaches were demonstrated in Figure 7. This induction of phenolic compound accumulation in the initial stage leads to a continuous increase in content, followed by the oxidation of the total phenolic content by polyphenol oxidase [44], resulting in a reduction in soluble polyphenol content and antioxidant properties; our findings are consistent with this viewpoint. The total phenolic content has a good correlation with free radical scavenging capacity [45]. When the number of hydroxyl groups in phenolic compounds increases, their antioxidant activity becomes more muscular. Phenolic substances and antioxidant activity can be used to evaluate the quality of fresh-cut nectarines. Another finding regarding the 1-MCP pre-cutting treatment of fresh-cut celery is that it can successfully postpone the levels of phenols [37]. Phenolic compounds are rapidly oxidized to quinones through the catalytic action of PPO, which then quickly polymerize to form dark brownish-black melanin-like compounds [46]. Meanwhile, ASA could reduce the formed quinone instantly to the original substrate (catechol) [42]. Furthermore, the reduced formation of quinone compounds in fresh-cut nectarines treated with 1-MCP also contributed to the alleviation of browning, consistent with the observations in the images. Therefore, the inhibition of PPO and POD activity by 1-MCP significantly suppressed the oxidation of phenolic content, ultimately leading to the inhibitory effect on soluble quinones in fresh-cut nectarines. These results suggest that 1-MCP inhibited PPO and POD activity as well as the metabolism of phenolic compounds, delaying enzymatic browning in fresh-cut nectarines.

The content of H_2_O_2_ and O_2_^•−^ generally accumulates during the mechanical damage process caused by fresh-cutting, which may alter the cell membrane structure and impede regular physiological metabolism [47,48]. MDA can affect the structure of the cell membrane and interfere with normal physiological metabolism. Studies have indicated that a higher accumulation of ROS results in an increase in MDA concentration, and MDA production also increases with the increase in membrane structure damage and integrity loss [49]. 1-MCP may inhibit the peroxidation reaction of membrane fatty acids, reduce the functional disorders of the membrane, and subsequently reduce the production of ROS, such as H_2_O_2_ and O_2_^•−^, and inhibit the production of MDA [50]. This is consistent with the decrease in the concentration of MDA and lower concentrations of H_2_O_2_ and O_2_^•−^ in this study. Previous studies have shown a relationship between the TPC and the activity of quenching DPPH radicals [51]. In our results, there was a significant increase in the DPPH scavenging ability in nectarines treated with 1-MCP, coinciding with the result in another report [52], which is related to the reduced accumulation of MDA and ROS. The 1-MCP pre-cutting treatment demonstrated lower POD activity, which can reduce the oxidative reactions induced by mechanical damage, decrease reactive ROS levels, and slow down the browning rate of fruits and vegetables [53]. The 1-MCP pre-cutting treatment can improve the antioxidant properties and decrease membrane lipid peroxidation in the treated fresh-cut nectarines by enhancing the high DPPH scavenging ability and inhibiting the generation of ROS. MDA is an important product in membrane lipid peroxidation [54]. The degree of browning in nectarines is also influenced by the damage to the cell membrane. It is possible that mechanically damaged nectarines lead to ROS accumulation, which causes severe damage to the cell membrane and aggravates the browning degree [55]. High levels of antioxidants and intact cell membranes were demonstrated by the 1-MCP pre-cutting treatment, which reduced the degree of browning in nectarines.

Aldehydes and alcohols are the main volatile substances. Aldehydes were the most abundant aromatic compounds tested in fresh-cut nectarines [31]. Fruit tissues during storage produce aldehydes through the metabolism of amino acids through fatty acid oxidation [56]. Through the action of associated enzymes, a number of alcohols, aldehydes, and esters might be linolenic and linoleic acid-derived secondary lipid metabolites [57,58]. Significant changes in aldehyde content were observed between the control and 1-MCP [19]. To prevent accumulation in mature fruits, aldehydes are converted into alcohols and lipids [59]. Some reports also pointed out that major aldehydes decreased gradually during shelf storage [60,61]. The esters give a pleasant peach flavor, indicating fruity and floral notes [62,63]. According to our findings, esters in the 1-MCP were first suppressed before progressively recovering over storage. Because aroma biosynthesis depends on ethylene, the data shown here suggest that the 1-MCP pre-cutting treatment depressed esters [64]. The 1-MCP pre-cutting treatment has been shown to be closely related to the production of intense aroma due to its inhibition effect on ethylene. The breakdown of polysaccharides provides carbohydrate and metabolic precursors to accelerate cellular metabolic activities. 1-MCP can inhibit the conversion of polysaccharides to volatile compounds, such as alcohols and esters [65]. The fruity smell of peaches is caused by terpenoids [66]. Similar to Wang et al. [19], the aroma was first inhibited after 1-MCP pre-cutting treatment and then gradually recovered, as evidenced by the change in linalyl acetate content according to our data.

In conclusion, ethylene regulates the volatile compounds involved in the biosynthesis of terpenoids and esters as well as the synthesis of volatile compounds in fresh-cut nectarines. Additionally, compared with the control, 1-MCP had lower aromaticity on day 8, probably due to the consistent ethylene release of fresh-cut nectarines by 1-MCP, which delayed the shelf life of fresh-cut nectarines, so that the aroma components were not completely released. These findings showed a correspondence between the GC-MS and E-nose analyses.

## 5. Conclusions

In this study, the whole nectarine fruit was treated with 1-MCP before mechanical damage, which could effectively prevent the browning and quality degradation of fresh-cut nectarines. The oxidation of phenolic compounds is inhibited by promoting the activity of POD and PPO. The mechanism demonstrated that the anti-browning effect may be the result of increased enzyme activity control, providing an alternative approach for anti-browning technology. It was also found that the 1-MCP pre-cutting treatment delayed the increase in MDA and ROS content, the quality deterioration of fresh-cut nectarines via antioxidant properties, and the inhibition of lipid peroxidation. Treatment with 1-MCP maintained cell membrane integrity and cellular ultrastructure examination. Moreover, the 1-MCP pre-cutting treatment can promote the formation of aroma and improve volatile compounds. The browning value of the 1-MCP pre-cutting treatment was 29.95% lower than the control on day 10, and the fresh-cut nectarines could still maintain the characteristic flavor, while the flesh maintains its firmness. In conclusion, 1-MCP can improve the quality of fresh-cut nectarines. It filled the gap that there were few studies on the color and aroma of fresh-cut nectarines after the 1-MCP pre-cutting treatment.

## Figures and Tables

**Table 1:** Classification of sensory properties of fresh-cut nectarines.

Project	Attributive Classification
aroma	Peach odor, grass odor, flower flavor
taste	Sweetness, sourness, bitterness
texture	Consistency, mealiness, juiciness
color	Flesh color, color uniformity

**Table 2:** Relative concentration of volatile substances in fresh-cut nectarines.

Compound	0 d	4 d—Control	4 d—1-MCP	8 d—Control	8 d—1-MCP
5,9-Undecadien-2-one, 6,10-dimethyl-, (E)-	-	0.175 ± 0.008 c	0.226 ± 0.016 b	0.232 ± 0.061 b	0.294 ± 0.035 a
Furan, 2-pentyl-	-	-	0.162 ± 0.004 b	0.163 ± 0.007 b	0.189 ± 0.016 a
Acetic acid, hexyl ester	0.110 ± 0.002 c	0.21 ± 0.005 d	0.154 ± 0.009 c	0.325 ± 0.009 a	0.294 ± 0.014 b
Ethyl 4-hydroxymandelate	0.019 ± 0.004 d	0.064 ± 0.015 c	0.044 ± 0.006 c	0.176 ± 0.006 a	0.128 ± 0.005 b
linalyl acetate	0.325 ± 0.018 c	0.599 ± 0.036 b	0.433 ± 0.035 bc	1.162 ± 0.124 a	1.171 ± 0.152 a
2-Hexyn-1-ol	0.667 ± 0.058 a	0.254 ± 0.009 d	0.474 ± 0.01 c	0.584 ± 0.035 b	0.574 ± 0.057 b
2-Octanol, (R)-	1.556 ± 0.125 c	2.275 ± 0.148 b	1.695 ± 0.012 c	3.181 ± 0.205 a	2.908 ± 0.188 a
3-Hexen-1-ol, acetate, (E)-	0.085 ± 0.002 a	0.223 ± 0.016 b	0.165 ± 0.015 c	0.762 ± 0.065 a	0.251 ± 0.015 b
2-Hexen-1-ol, acetate, (E)-	0.062 ± 0.003 c	0.155 ± 0.015 ab	0.183 ± 0.019 a	0.141 ± 0.017 b	0.167 ± 0.025 ab
Hexanal	44.664 ± 2.943 a	38.227 ± 3.005 bc	42.666 ± 2.875 ab	32.944 ± 2.573 c	36.225 ± 2.649 c
2-Hexenal	37.557 ± 2.758 a	33.159 ± 2.874 ab	35.175 ± 2.546 a	22.654 ± 2.453 c	29.867 ± 2.047 b
Benzaldehyde	0.676 ± 0.055 d	0.573 ± 0.055 d	1.996 ± 0.009 a	1.453 ± 0.125 b	1.224 ± 0.012 c
Nonanal	2.293 ± 0.159 a	2.098 ± 0.188 a	2.298 ± 0.165 a	0.737 ± 0.055 c	1.076 ± 0.023 b
2-Heptenal, (Z)-	-	0.072 ± 0.002 b	0.075 ± 0.007 b	0.151 ± 0.018 a	0.083 ± 0.007 b
Decane	1.097 ± 0.046 a	0.856 ± 0.035 ab	0.925 ± 0.082 ab	0.446 ± 0.054 c	0.584 ± 0.427 bc
Octanal	-	0.066 ± 0.002 c	0.152 ± 0.050 b	0.163 ± 0.016 b	0.325 ± 0.025 a
trans-2-Nonenal	-	0.263 ± 0.056 a	0.339 ± 0.246 a	0.331 ± 0.025 a	0.279 ± 0.007 a
Cyclotrisiloxane, hexamethyl-	0.099 ± 0.006 b	0.176 ± 0.032 a	0.193 ± 0.017 a	0.158 ± 0.025 a	0.175 ± 0.004 a
Cyclopentasiloxane, decamethyl-	1.371 ± 0.125 d	7.536 ± 0.764 a	3.057 ± 0.042 c	4.032 ± 0.059 b	6.817 ± 0.718 a
Decane	-	0.142 ± 0.016 c	0.639 ± 0.033 ab	0.738 ± 0.160 a	0.565 ± 0.037 b
Cyclohexasiloxane, dodecamethyl-	0.396 ± 0.017 d	3.329 ± 0.264 a	1.166 ± 0.104 c	2.624 ± 0.157 b	3.228 ± 0.068 a
Cyclotetrasiloxane, octamethyl-	-	1.659 ± 0.103 a	1.653 ± 0.137 a	1.290 ± 0.135 b	1.758 ± 0.197 a
Octadecane, 6-methyl-	0.117 ± 0.004 b	0.133 ± 0.009 a	0.095 ± 0.007 c	-	0.098 ± 0.008 c
Styrene	0.353 ± 0.025 a	0.078 ± 0.004 c	0.172 ± 0.008 b	-	-
β-Ocimene	0	0	0	0.117 ± 0.016 a	0.1 ± 0.065 a
o-Cymene	0.055 ± 0.003 d	0 ± 0	0.068 ± 0.003 c	0.09 ± 0.003 a	0.075 ± 0.006 b
Azulene	0.132 ± 0.018 b	0.478 ± 0.012 a	0.473 ± 0.016 a	0.455 ± 0.025 a	0.468 ± 0.015 a
Estragole	0	0	0.072 ± 0.005 b	0.093 ± 0.006 a	0.019 ± 0.002 c
Linalool	0.469 ± 0.044 a	0.299 ± 0.026 b	0.465 ± 0.015 a	0.325 ± 0.005 b	0.234 ± 0.005 c
D-Limonene	0.453 ± 0.019 d	0.853 ± 0.057 b	0.614 ± 0.026 c	0.837 ± 0.035 b	0.923 ± 0.008 a

Note: Data are presented as means ± SD. Relative concentration (%), 2-octanol (7.90 mg/L) as an internal standard. Different letters aside indicate statistically significant differences at *p* < 0.05.

## Data Availability

The original contributions presented in this study are included in the article. Further inquiries can be directed to the corresponding author.
